# To die well: the phenomenology of suffering and end of life ethics

**DOI:** 10.1007/s11019-019-09914-6

**Published:** 2019-08-28

**Authors:** Fredrik Svenaeus

**Affiliations:** Centre for Studies in Practical Knowledge, Södertörn University, Stockholm, Sweden

**Keywords:** Phenomenology, Suffering, Narrative, Dying, Palliative care, Euthanasia

## Abstract

The paper presents an account of suffering as a multi-level phenomenon based on concepts such as mood, being-in-the-world and core life value. This phenomenological account will better allow us to evaluate the hardships associated with dying and thereby assist health care professionals in helping persons to die in the best possible manner. Suffering consists not only in physical pain but in being unable to do basic things that are considered to bestow meaning on one’s life. The suffering can also be related to no longer being able to be the person one wants to be in the eyes of others, to losing one’s dignity and identity. These three types of suffering become articulated by a narrative that holds together and bestows meaning on the whole life and identity of the dying person. In the encounter with the patient, the health-care professional attempts to understand the suffering-experience of the patient in an empathic and dialogic manner, in addition to exploring what has gone wrong in the patient’s body. Matters of physician assisted suicide and/or euthanasia—if it should be legalized and if so under which conditions—need to be addressed by understanding the different levels of human suffering and its positive counterpart, human flourishing, rather than stressing the respect for patient autonomy and no-harm principles, only. In this phenomenological analysis the notions of vulnerability and togetherness, ultimately connecting to the political-philosophical issues of how we live together and take care of each other in a community, need to be scrutinized.

## Introduction

Questions of physician-assisted suicide and euthanasia are complicated, not only because they involve a health care professional doing something that is apparently opposite to what she is normally supposed to do, namely helping to kill or killing which stands in contrast to curing diseases and saving lives. These questions are complicated because it can sometimes be very hard to judge whether a life situation is truly hopeless and undignified—meaning there is nothing that can be done about the severe suffering—or whether there are still solutions to be found that would lead to a life worth living for the person who wants to die. Despite the progresses in palliative care, there still seem to be some cases in which pain relieving therapies do not work to a sufficient degree, thus leaving the patient in intolerable pain. Even trickier, though, are the cases in which the perceived intolerable suffering consists not in physical pain but in being unable to do basic things that are considered to bestow meaning on one’s life—like having a good meal, going for a walk, reading the newspaper, or joining in a discussion with friends. The suffering can also be related to no longer being able to be the person one wants to be in the eyes of others, to losing one’s dignity and identity. Such fundamental values become articulated by a narrative that holds together and bestows meaning on the whole life and identity of the person in question. In this paper, I will develop a phenomenological account of suffering based on concepts such as mood, being-in-the-world and core life value that, I will argue, better allows us to understand and evaluate the hardships associated with dying and there through may assist health care professionals in helping persons to die in the best possible manner. A phenomenological account of suffering proceeds from the first-person perspective of the patient, rather than the third- (or, rather, non-) person perspective of medical science. In the encounter with the patient, the health-care professional attempts to understand the first-person suffering-experience of the patient in an empathic and dialogic manner (or, at least, he or she should do so), in addition to exploring what has gone wrong in the patient’s body (Svenaeus 2000, part 3). I will claim that the combination of caring and empathic listening with adequate medical investigations and therapies is arguably the best way to help suffering, dying persons.

## What matters in human life to persons and the phenomenology of suffering

In his celebrated book *Being Mortal: Illness, Medicine, and What Matters in the End* Atul Gawande writes:

People with serious illness have priorities besides simply prolonging their lives. Surveys find that their top concerns include avoiding suffering, strengthening relationships with family and friends, being mentally aware, not being a burden on others, and achieving a sense that their life is complete. Our system of technological medical care has utterly failed to meet these needs, and the cost of this failure is measured in far more than dollars. The question therefore is not how we can afford this system’s expense. It is how we can build a health care system that will actually help people achieve what’s most important to them at the end of their lives. (Gawande 2014, p. 155)

Reading through Gawande’s well researched book and also taking into account other studies of what matters in the end of life and what type of situations can lead patients to express a wish to die (Müller-Busch 2015), we end up with a list like this:

Intolerable pain. Not being able to breathe. Constant nausea. Leaking urine and faeces. Not being able to do basic things, such as eating, going to the toilet, reading, and moving around. Becoming dependent upon or a burden to close others. Not having a place and purpose in the world any more. Losing one’s memory and sanity. No longer being in control. Losing one’s dignity.

What are these painful experiences about? What do they consist in? I would like to propose that, from a phenomenological point of view, they could all be viewed as different *moods* of *suffering* involving different *levels* of what the phenomenologist refers to as a being-in-the-world:

Suffering is an alienating mood overcoming a person and engaging her in an embodied struggle to remain at home in the face of the loss of meaning and purpose in life. It involves painful experiences at different levels that are connected through the suffering-mood but are nevertheless distinguishable by being primarily about (1) my embodiment, (2) my engagements in the world together with others, and (3) my core life values. (Svenaeus 2014, p. 413)

That feelings—I am using this term in an all-encompassing sense—in the form of moods are not only bodily sensations but, more importantly, make for meaningfulness by opening up a life world of objects, actions, thoughts, communication, and so on, is a thematic developed by phenomenologists such as Max Scheler, Martin Heidegger, and Jean-Paul Sartre (Freeman 2014; Solomon 2006). Different things show up in the life world because of the mood a person is in, and they do so through a certain background meaning structure, often referred to by phenomenologists as the person’s being-in-the-world. Human animals have a much richer world than other animals because the things that show up to them through moods are interwoven in patterns of meaning that have developed into what we might call a *culture*: a system of human-made significance that is articulated and communicated in and through a language.

Why are the phenomenological issues of mood and being-in-the-world important from the perspective of suffering and end of life ethics? Because they provide clues for understanding how physical suffering is connected to the other types of suffering which feature in the list of what may lead persons to express a wish to die: frustrated life plans and broken narratives. Many other things than physical pains can make a person suffer: to not get what you want, to get what you really do *not* want, to not become who you want to be, or to become who you really do *not* want to be, for instance. Wishes and strivings for certain goals in life are surely also forms of feelings, but as *emotions* they include, in contrast to pain, specific thoughts. They are ways of presenting not only the whole world but also specific states of the world as what is to be desired by the person who has them (Goldie 2000). The thoughts in question can be more or less conscious to the person having the emotions. The ways we live and embody ideals and values in life are not always very well reflected but rather subconscious. Having said this, how are we to think about a “life plan”, or a “life narrative”, and the way they can be frustrated for a person? How explicit are the goals we set for ourselves in our lives?

The most intriguing part of the phenomenology of suffering is perhaps the way a person’s suffering is both determined and potentially changeable by way of the *core life values* she embodies. What does this mean? If I am a concert pianist, the sudden painful inability to move my little finger is much more important to me than if I am a librarian. In the same way, finding out that my wife has been having an affair is much more devastating if I believe in life-long faithful marriages than if I believe that the ideal of monogamy is a destructive illusion. The moods we live in embody such life priorities and evaluations by the way they make things in our life appear as more or less *significant* to us. Charles Taylor, in *Sources of the Self*, analyses the way our personal beings, are built up by way of such evaluations. Most important are those priorities he calls “strong evaluations”, evaluations about the things that makes a human life worth living beyond satisfying the basic needs of food, drink, sleep, safety, love and sex (Taylor 1989, 4 ff). The moral philosopher Ronald Dworkin calls the same things “critical interests” (1994, 199 ff). These strong evaluations concern moral matters: what responsibilities I have for the life and flourishing of other persons. They also, however, concern questions about what a *good life* means for me and how I attain *self*-*respect in the eyes of others*:

To understand our moral world we have to see not only what ideas and pictures underlie our sense of respect for others but also those which underpin our notion of a full life. And as we shall see, these are not two quite separate orders of ideas. There is a substantial overlap or, rather, a complex relation in which some of the basic notions reappear in a new way. This is particularly the case for what I called above the affirmation of ordinary life. In general, one might try to single out three axes of what can be called, in the most general sense, moral thinking. As well as the two just mentioned—our sense of respect for and obligations to others, and our understandings of what makes a full life—there is also the range of notions concerned with dignity. By this I mean the characteristics by which we think of ourselves as commanding (or failing to command) the respect of those around us. (Taylor 1989, pp. 14–15)

In tracking the origins of the modern concept and experience of selfhood, Taylor, in addition to this preliminary outline of the territory of strong evaluations, spends considerable time articulating the importance of *self*-*expression* for our ways of being constituted as persons (selves) in the modern era. Protestantism and romanticism are his major sources in stressing the importance of *spelling oneself out* by way of a form of creative work (Taylor 1989, p. 374). The artist, the genius of the Romantic era, creating her works of art and herself by making her inner nature visible to us in the form of a painting or a poem, is exemplary in this regard. From this image it is not a very long leap to a model of the self—the person—as constituted by a life narrative, a model we find in contemporary cultural theory and medical ethics (Schechtman 1996). Taylor’s strong-evaluation idea about what essentially matters to us in life and how we may flourish is also consonant with research in developmental psychology about how we attain a sense and concept of selfhood together with and in the eyes of others (Rochat 2009, 86 ff). Our feelings of who we are and what matters to us in life are to a very large extent dependent on the way we connect to others and their views on us. The story of a human life is from the very beginning a narrative that attains meaning for a person in the eyes of others.

The idea that a person (a self) is a narrative obviously has to be interpreted in some metaphorical way to make sense (see many of the essays in Gallagher 2011). Human lives are not stories, written or told, in the strict sense of the word. The life of a person, however, clearly has a temporal structure by including a beginning and an end, and also a cohering structure in the way that the life must make, at least minimal, sense to the person in question and to others attempting to understand her. When we strive to understand life events involving persons we turn to stories. The narrative structure is where the cohesiveness of a human life comes from: it is not enough to have temporal continuity; one also needs to develop a narrative to explore and to show who one is (Goldie 2012; Ricoeur 1992). The question of personal identity in this extended sense is connected to the core life values we identify with. The most important values as regards self-identity are the ones Taylor identifies as demanding strong interpretation: values regarding the treatment of others; values regarding the content of a good life; and values regarding the identification of oneself as someone worthy of respect in the eyes of others (Taylor 1989; see also Taylor 1991). These three zones of core life values are interconnected, and they demand, at least to some extent, self-reflection. But core life-narrative values do not come about only through philosophical reflection; they become embodied by living in the world and sharing it with others from the very start. Strong evaluations are always dependent upon a life form, a horizon of attuned understanding that one has grown into through the support and influence of others and they can thus be more or less implicit or explicit for a person. Core life values are, nevertheless, always core life-*narrative* values, because they are only possible to comprehend and/or formulate by way of stories about a person’s life (Goldie 2012, Chapter 6). A human life is not a narrative but rather it is imbued with reason and coherence through stories that can be more or less true to the life of the person they are about (Goldie 2012, Chapter 7).

## The case of Ivan Ilyich

In order to exemplify and concretise the phenomenological argument about suffering, let us now turn to the short story about Ivan Ilyich, often used to discuss issues surrounding dying and suffering in medical ethics (Tolstoy 2015). Ivan is a fairly successful lawyer living a seemingly happy life with his family and friends in Saint Petersburg when illness, probably some sort of intestinal cancer, hits him:

The pain in his side oppressed him, and seemed to be constantly getting worse; it became a continuous pain, and the taste in his mouth became stranger and stranger. It seemed to him that his breath smelt disgusting, and his appetite got worse and he felt weaker all the time. He could not deceive himself: something new and terrible was happening to him, something so important that nothing that had ever happened to him in his life had been more important. And he was the only one who knew; those around him didn’t understand, or didn’t want to understand, and they all thought that everything was going on as before. That was what tormented Ivan Ilyich more than anything. His family—above all, his wife and daughter, who were in a positive whirl of engagements—understood nothing, as he could see; they were vexed that he was so morose and demanding, as if it was his fault. They tried to hide it, but he could see that they found him a nuisance. (Tolstoy 2015, p. 181)

The doctors of this time—the 1880s—were not able to do much about cancer, especially not if it had metastasized, but the worst thing for Ivan is not that he suspects the doctors do not have a clue about what causes his abdominal pain (a virtual line-up of famous and expensive physicians are consulted as his condition deteriorates). The worst thing is that they, just like his family and friends, neither see nor understand *him* and his suffering:

The doctor said: such-and-such and so-and-so indicate that within your body you have such-and-such and so-and-so; but if the investigations of such-and-such and so-and-so fail to confirm this, then we still have to conclude the presence of such-and-such and so-and-so instead. But if we suppose such-and-such, then, etc. Ivan Ilyich was only interested in one thing: was his condition dangerous or not? But the doctor ignored this improper question. From the doctor’s point of view, such a question was pointless and could not be discussed; the only thing that mattered was to weigh up alternative probabilities – a wandering kidney and a disorder of the blind gut… From the doctor’s summing up, Ivan Ilyich came to the conclusion that things were bad; that the doctor didn’t care, and probably nobody else did either, but for him they were bad. And this conclusion struck Ivan Ilyich painfully, making him feel very sorry for himself and angry with this doctor who was so indifferent to a matter of such importance. But he said nothing. He stood up, laid his money on the table, and sighed. (Tolstoy 2015, p. 178)

The medical-scientific abilities and skills involved in understanding such-and-such and so-and-so have advanced immensely since the times of Ivan Ilyich, but despite this, many patients and physicians testify that the tendency to neglect the suffering and dying person for all his diseases is still in place (Bishop 2011; Cassell 2004; Gawande 2014). This is so for several reasons: the dominance of the scientific perspective in contemporary medicine; the tendency to divide the investigation and treatment of a patient between different medical specialities and professionals; the unwillingness to address matters concerning impendent death in a discussion with the patient because this will involve anguish and terror; and finally the wish to focus on keeping the patient alive, since for many physicians death is the ultimate disaster and failure to be avoided.

Doctors are supposed to save lives, not end them, but in some situations they are faced with the choice of treating a disease that is killing the patient or attempting to mitigate his suffering. Currently, in such cases, when further treatment of the disease will only prolong life marginally and it will actually mean increased suffering for the patient, the recommendation by experts is increasingly to focus on palliation rather than fighting disease. Patients have the right to choose between various options that doctors judge to be medically feasible and advisable, but before presenting such choices the professionals should take care to empathically understand the suffering persons they are facing and what their main issues are (Gawande 2014). The heroic imperative of “doing everything possible” in all situations, and putting one’s faith in a medical science that will soon be able to treat every disease, has vanished as it has become obvious that, in some situations, advanced treatment possibilities and technologies can intensify and prolong a patient’s suffering rather than the other way around.

Doctors have become incomparably more successful in mitigating the kind of bodily pains that Ivan Ilyich suffers from in the novel, not least the pain he endures the last three terrible days of his life:

It was from that moment that the screaming began, which was to continue uninterrupted for three days, a screaming so dreadful that even through two closed doors it was impossible to hear it without horror. In that moment when he answered his wife, he realized that he was lost, there was no return, the end had come, the end of everything, and yet his doubt had still not been resolved, it still remained a doubt. “O! O! O!” he screamed in different intonations. He had begun by crying “No!”, and so went on, continuing with the sound “o”. (Tolstoy 2015, p. 207)

Are contemporary doctors also better at understanding the core life-narrative values of their patients than Ivan’s doctors (as well as family members and friends) were? Not necessarily; the skills of empathy, dialogue, and narrative understanding have not been focused upon in modern medicine until fairly recently, and in many settings they remain more or less absent, overshadowed by the focus upon medical science and diseases of the body. To some extent, the medical-scientific successes of the last century have even ignored “the art of medicine’, a tradition which some doctors in the times of Ivan Ilyich knew and practiced (not the ones he encountered, though) (Svenaeus 2000, part 1).

## Palliative care, physician assisted suicide and euthanasia

Palliative care is a speciality that is increasingly focused upon in modern medicine, and we can hope that doctors will become even better at treating the pains we often suffer towards the end of our lives. However, as I have tried to argue above, suffering is not only about physical pain but also about what we are able to do in the world and who we are able to be there in the company of others. Physicians like Jeffrey Bishop, Eric Cassell and Atul Gawande all stress these additional suffering domains in their attempts to understand the pains and hardships of dying persons (Bishop 2011; Cassell 2004; Gawande 2014). Human life is a “being-towards-death’, as Heidegger puts it in *Being and Time*, and this means that death is not only a physiological event at the end of our life but a relationship to our own ending that we potentially face all the time (Aho 2016; Carel 2008; Heidegger 1996, 235 ff). The meaning of my life narrative and the core life values I more or less consciously embody are inseparable from the beginning and end of my life. A story always has a beginning and an ending; this is part of what makes it a story with a certain plot. And a miserable ending, at least if it is a long and disruptive one, can change the meaning of the whole life story if a person becomes severely alienated concerning the ways she lives and looks upon herself in the more or less imagined eyes of others (Dworkin 1994, 199 ff).

When we become old our bodies inevitably display a vulnerability we have actually been suffering from ever since we were born (MacIntyre 2001). Human bodies are weak and rather defenceless ever from the start in being susceptible to countless forms of injuries and diseases. “Transhumanists” dream of an age when we will no longer have to die, because doctors and other scientists will be able to fix or replace our ageing body (parts) (O’Connell 2017). However, for a foreseeable future we will have to live with our vulnerable condition, which means that ageing inevitably comes with more illness suffering and the kinds of alienation that follow in its track. We adapt to this increasingly vulnerable and weak condition with the more or less spontaneous change of lifestyle that often commences in growing old. Old people live slower and more cautious lives; they become less focused upon doing new things and treasure relationships with people they already know. To become older means embodying a life narrative that is coming to a close, and this is not necessarily a bad or sad thing.

In using the expression “embodying a life narrative” I literally mean a person’s lived embodiment as the central aspect and way of existing in a life world. Ways of embodiment change with age, and this is also the reason persons modulate or change the preferred life projects from which they derive their core life-narrative values. We generally become less physically active and more thoughtful as we grow older; we often care less about our shortcomings and appreciate the things we are still able to do. In this manner we can escape alienation and even become more at home with ourselves in getting closer to the end of our life. But in some cases, the changes brought on us by diseases and other sad life events are possibly too severe or tragic to allow for changed life priorities. We feel the suffering is too much to bear and live with, and we would rather die than survive in this condition and situation if nothing can be done about it.

If faced by the choice of either living the last 3 days of our lives as Ivan Ilyich did or receiving a lethal injection at the beginning of day one that would kill us painlessly, arguably a vast majority would choose the latter. But doctors are better at treating pain today than they were in the 1880s, and many argue that we do not need the option of the lethal injection to live tolerable and dignified lives to our very end. This is probably so in most cases, but there still seem to be some cases in which palliation does not work to a sufficient degree, thus leaving the patient in intolerable pain (Müller-Busch 2015, p. 185). Even trickier, though, are the cases in which the perceived intolerable suffering consists not in physical pain but in being unable to do what is seen as the things that bestow meaning on one’s life (Müller-Busch 2015, p. 185). Is it always possible to adapt by changing one’s fundamental goals in life, or are some changes beyond what is reasonable to expect, especially in consideration of persons who will soon die and do not have much time in which to realize their changed life priorities? Clinical empathy and medical hermeneutics demand an attempt to understand the whole life situation and identity of the patient, especially in cases of severe, chronic, and terminal suffering. What does the patient’s life look like and what makes it worth or not worth living? What does he fear the most and why is this the case? Only through empathically asking such questions and interpreting the responses can doctors help patients to die well in end-of-life care (Gawande 2014, Chapters 7 and 8).

The many ways persons suffer in end of life care surveyed above provide arguments for allowing physician assisted suicide and/or euthanasia (PAS/E) by law, as has happened in a numberof Western countries during the last 25 years (the Netherlands, Belgium, Luxemburg, some states in the United States, and Canada) (Birnbacher and Dahl 2008; Cholbi and Varelius 2015). These sufferings, as we have seen, do not only concern bodily ailments but also cherished activities and core life values. Such life values become articulated by a narrative that holds together and bestows meaning on the whole life and identity of the person in question (Baker 2000; Ricoeur 1992; Schechtman 1996). If, in view of a chronic medical condition that plagues her and will soon lead to her inevitable death, a person finds her current situation incompatible with being the kind of person she has become and wants to be in and through her life narrative, this is a very strong argument for allowing her to die (Dworkin 1994, pp. 235–236).

Such cases could also include situations of advance directives, as they are called, in which persons have stated that they do not want to be kept alive by means of feeding tubes or ventilators should they end up severely demented or enter a vegetative state. However, allowing someone to die is not the same thing as assisting a person in taking her life, much less killing her; if the latter two actions are to be allowed we minimally need the person to be able to asses her present condition in a realistic way and ask for this help. If such conditions are fulfilled, the phenomenological perspective on personhood and suffering in medicine, in my view, does not rule out that some measures taken to mitigate or avoid severe suffering for a patient may include at least assisting him in taking his life. But how should such a moral conclusion reached by way of paying attention to the potential sufferings of dying persons be cached out regarding the implementation of guidelines and laws regulating what dying patients have the right to ask for and health care personnel assisting them have the right to do? This is the complicated question to which I now turn.

## Vulnerable suffering persons and existential politics

Matters of PAS/E—if it should be legalized and if so under which conditions—need to be addressed by understanding human suffering and its positive counterpart, human flourishing, rather than comparing and balancing ethical principles such as respecting patient autonomy versus not harming the patient (Beauchamp and Childress 2013). In such a phenomenological analysis the notion of *togetherness*, ultimately connecting to the political-philosophical issues of how we live together and take care of each other in a community, should be scrutinized. A person can only flourish, living a life in which she develops her own nature and prospects, in a community *together with others*, in a community based on values that reinforce a mutual responsibility for the common good (Arendt 1998). Consequently, euthanasia issues are ultimately authenticity and community issues rather than autonomy and not-harming issues only.

If we return to the third level of suffering surveyed above, we find the strongest arguments for, as well as against, allowing PAS/E by way of law. The reason the third level of suffering not only involves the strongest arguments for but also against allowing PAS/E is that the narrative identity, which can be felt to be impossible to live with in a situation that is perceived as an undignified condition, is a self-respect *in the eyes of others* (Taylor 1989, pp. 14–15). Persons constitute their value and worth in relationship to others, and if—as is often the case when persons became unable to take care of themselves in advanced age—they feel they *should* wish they were dead, they might say so, not only to their relatives, but also to their doctors. Increased patient autonomy has undoubtedly been one of the most important developments in late modern medicine—inter-nested with the rise of medical ethics as such in the 1960s and 1970s—but a too-narrow view of the person as a rational decision maker devoid of context and narrative is an easy and potentially dangerous way out of taking professional responsibility for the patient and his well-being (Halpern 2001; Jonsen 1998).

Drafting and passing laws concerning PAS/E could be considered as political *actions* in Hannah Arendt’s terminology (Arendt 1998). They involve performances on a public stage in which debating and passing judgements concerning the essence of human goods and rights are enacted. For Arendt, it is crucial that political performance is a way of *showing ourselves* in the sense of displaying who we are in front of others and exploring what views we may come to hold together with them by way of discussion. Actions in the political space are not only about defending human rights and just distribution of resources, it is also a matter of preventing existential questions from being swallowed up by issues of utility and productivity. It is a way of finding out who we are and what a human life worth living consists in. Arendt’s political philosophy finds its roots in the phenomenology and existential philosophy of Martin Heidegger and Karl Jaspers in which the question of human flourishing, in the sense of finding out who you/we are and what you/we want to live for, looms large (Loidolt 2018). The risk Arendt perceives and identifies in modern politics is precisely that the meaning of human life is taken to concern *utility* rather than human flourishing. A successful human life is taken to be a *productive* life in the sense of being profitable for the society. Such views become incorporated in totalitarian systems in which individuals are no longer respected or protected if they do not contribute to the common good of the race (Nazism) or the class (communism), but they may also thrive in liberal democracies, in which politics becomes business-like and in which existential questions become private-life issues only (Arendt 1973).

In such liberal societies, each and every one has the right to flourish in her own way, provided she can find the resources for her life-project, but the political sphere does not provide the existential discussion in which different views on human goods can be seriously discussed and defended. Arendt’s prognosis is that in such a situation the economic productivity-ideology will outflank the attempts to show that human lives are meaningful in ways that cannot be reduced to a utility and pleasure calculus only. To return to the dilemmas of end-of-life suffering and PAS/E, the question if a person’s life is worth living may very well become strongly influenced by the utility-productivity paradigm. If you do not feel much pleasure but rather pain, if you are no longer able to do things that make your life meaningful, and even less contribute to the flourishing of others, your life may quickly look like a useless productivity drain, stealing time and resources from others. If I ended up like that, you hear the others (at least imaginatively) saying, I wish I had the guts to kill myself, and, if I did not, I wish someone else had the pity to do it for me.

The risk of institutionalizing PAS/E is that an increasing number of human suffering situations will be viewed as irrational in the sense that they should better be avoided by way of ending them rather than continuing them if we have the choice. Some moral philosophers infected by the utility paradigm even hold that most, or even all, human lives are not worth living in comparison with not existing (Benatar 2006). However, as the phenomenological analysis of suffering and flourishing shows, the meaning of human life is not to be found in any pleasure-pain calculus but rather in a framework that addresses meaningfulness in terms of embodied moods, shared being-in-the-world and narratives. To allow PAS/E by way of law could be viewed as an empathic act in view of empowering patients and making it easier to end useless suffering, but if the society in which the law is implemented does not cultivate solidarity bonds with suffering persons in need of care, the law may rather reinforce the rationality-productivity paradigm by way of which an increasing number of dying persons will find it harder to make any sense of the remains of their suffering-afflicted lives.

The risks of implementing laws allowing for PAS/E increases with totalitarian tendencies in a society on the one hand and/or rationalistic tendencies in a society on the other. Leaving the obvious risks of totalitarian societies aside (think of the euthanasia programs for getting rid of “unworthy life” in Nazi Germany), it should be noticed that the countries that have implemented PAS/E laws so far often are highly secularised as concerns the world views of their citizens. The standard way of explaining this is that religion provides a ban on suicide and mercy killing that prevents the institutionalization of PAS/E, but a different understanding is that a religiously dominated culture provides a sphere for addressing existential questions and finding meaning in life beyond the pleasure-pain calculus by way of personal belief and religious worship.

A common claim, that is hard to evaluate, is that euthanasia is performed on a regular basis by doctors also in countries in which it is not (yet) allowed. Apparently, there exists a rather large grey zone in which palliative therapies to relieve pain also have the (un)intended side effect of making the lives of suffering patients a few days or even weeks shorter than would otherwise have been the case (Warraich 2017, pp. 249–266). Palliative sedation is another method in use, which may have the side effect of shortening the life of the patient, and which I have not discussed in this paper. The down side of keeping euthanasia in the grey zone as a side effect of pain management is that suffering due to not being able to engage in the world together with others or living up to one’s core life values (level 2 and 3) are generally not taken into account if they are not accompanied by severe bodily pain (level 1). The advantage of doing so (not implementing laws allowing for PAS/E) is that the utility paradigm is not encouraged to infect our views on human flourishing and suffering.

A pragmatic compromise ensuring that doctors stay within the palliative dimension and are not assigned the executive role of ending lives would be the so-called Oregon model institutionalized in an increasing number of states in the USA. This model allows for physician assisted suicide, but not euthanasia, when a patient suffers from a disease that will end his life within 6 months. Two independent doctors must be involved in the evaluation of the medical condition (predicted death within 6 months) and ensure that the patient is mentally competent and does not suffer from a treatable psychiatric condition (e.g., depression) which affects the decision. The Oregon model has been in use since 1997 and statistics show that it is the cause of death in around 0.4% of the cases of total number of deaths in the states in which it has been applied. In comparison PAS/E is the cause of death in around 4% of the total number of deaths in the countries—Belgium, The Netherlands, Luxemburg—that have legalized the right to euthanasia (Smer 2017, pp. 92–97). The right to receive euthanasia in BeNeLux applies to persons who experience suffering assessed to be unbearable and impossible to treat by the doctors, and the right is not exclusive to end of life suffering or to somatic in contrast to psychiatric cases of illness. The BeNeLux model has been in use since 2002 and has led to a steadily increasing number of deaths by way euthanasia in the practicing countries. If this is a good thing or a bad thing depends on the evaluation of the individual cases, but the rather high numbers motivate the concerns I have voiced above about existentially unaware politics. On the other hand, one could make the argument that the legalizing of euthanasia is a perfect example of a highly aware existential politics, which does not shy away from the important questions on what kinds of human lives are worth living. However, the worry would still stand that the political discussion in the countries in question have not sufficiently considered the ways in which a person’s dignity and wish to live is dependent on how her life appears in the eyes of others when vulnerable and desperately in need of solidary assistance and support from fellow human beings. The phenomenological analysis developed in this article provides resources to identify these dimensions of human suffering in a systematic way and underline their importance in an existentially aware political discussion about the pros and cons of legalizing PAS/E.

## Conclusion

Medicine and end of life ethics could profit from a phenomenological theory of suffering in several ways. First, by acknowledging and becoming better in understanding the attuned, experientially-integrated multi-level character of suffering. Second, by better understanding how different levels of experience—embodiment, daily activities, core life values—could all be important and interconnected in mitigating (or possibly ending) suffering for a person. Third, by better understanding what wholeness and completeness may mean to a dying person, that is: what it means to have completed a human life, dying as the person one wants to have been, in one’s own eyes, and in the eyes of others. The phenomenological account of suffering as a multi-level experientially-integrated phenomenon could also be used to articulate a better informed existential-political argument about the implementation of physician assisted suicide and/or euthanasia. In this way phenomenology may assist and contribute to the analysis of medical ethical dilemmas associated with end of life suffering within the domains of health care and within society as a whole.
